# Supplementary material for: Two distinct conformational states define the interaction of human RAD51‐ATP with single‐stranded DNA
Source: EMBO J. 2018 Mar 5;37(7):e98162. doi: 10.15252/embj.201798162 (PMC5881629; doi:10.15252/embj.201798162)
Supplement: Supplementary file 1 — Appendix [file EMBJ-37-e98162-s001.pdf]

Appendix

Contents:

Appendix Figure 1	page 2-3
Appendix Figure 2	page 4
Appendix Figure 3	page 5
Appendix Figure 4	page 6
Appendix Figure 5	page 7-8
Appendix Table 1	page 9
Appendix Table 2	page 10

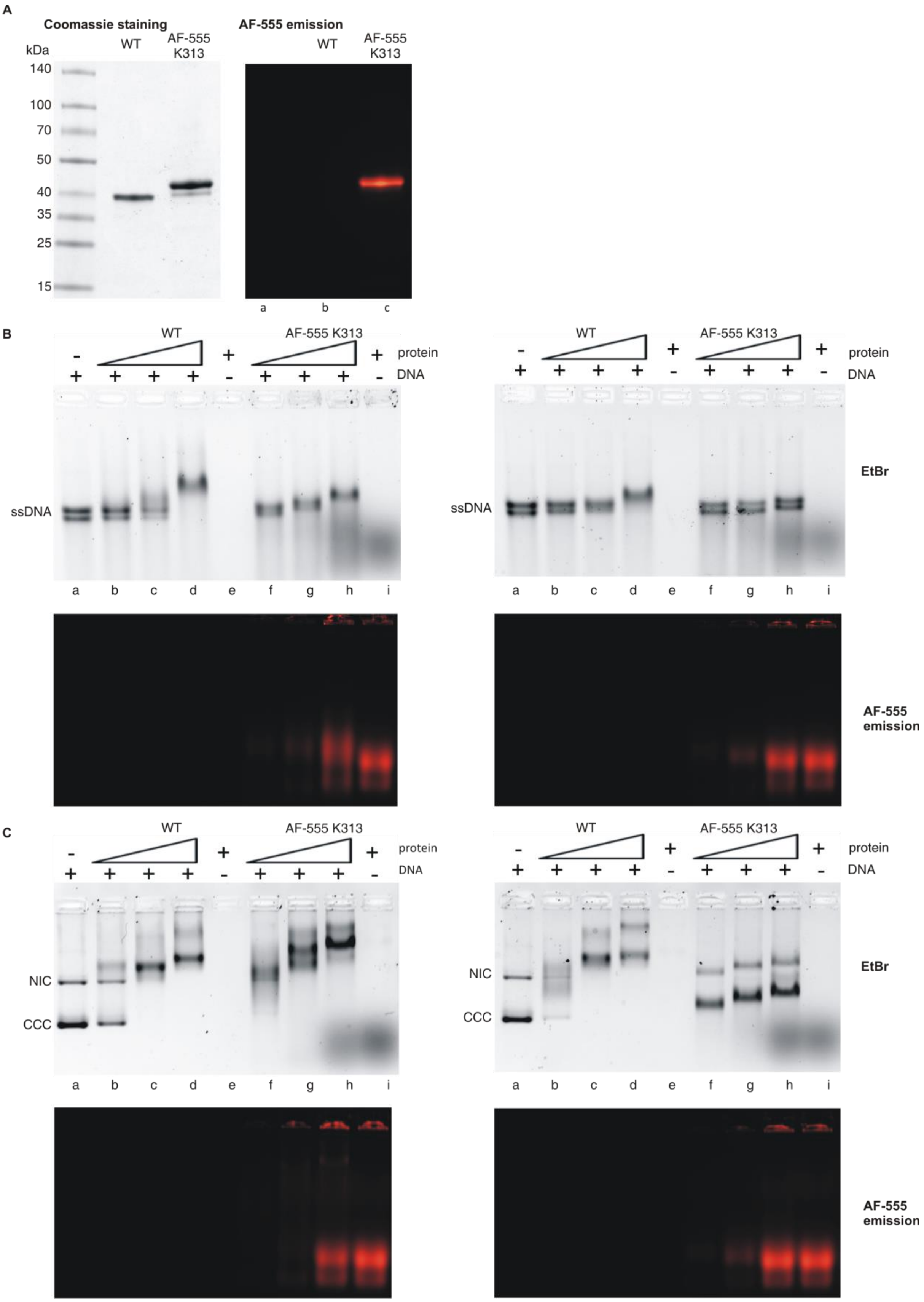

Appendix Figure 1: Purification and DNA binding of Alexa Fluor 555 labeled hRAD51 K313 compared to wild-type protein.

A SDS-PAGE of purified protein (0.75 µg) hRAD51 unlabeled wild-type (lane b), hRAD51 K313 C319S labeled on C31 with Alexa Fluor 555 (lane c). After electrophoresis the gel was analyzed with a ChemiDoc MP imaging system (BioRad, software Image lab vs 4.1) to specifically excite and detect emission of Alexa Fluor 555 (right panel). Next, the gel was stained with Coomassie Brilliant Blue R-250 to reveal all proteins and visualized by bright-field illumination (left panel). Lane a = molecular weight standard. AF = Alexa Fluor.

B, C Electrophoretic mobility shift assays showing the ssDNA (B) or dsDNA (C) binding activity of unlabeled hRAD51 wild-type and hRAD51 K313 C319S labeled with Alexa Fluor 555 in presence of Ca^{2+} (left panels) or Mg^{2+} (right panels). Each protein was incubated with ssDNA (PhiX174 virion ssDNA) or dsDNA (PhiX174 RFI dsDNA) (200 ng) in a 20 µl reaction volume containing 75 mM KCl, 55 mM Tris-HCl, pH 7.5, 0.25 mM EDTA, 0.25 mM DTT, 2.5% glycerol, 1 mM ATP, 2 mM $CaCl_2$ or $MgCl_2$ and increasing amounts of protein (0.5 µM, 1 µM, 2.5 µM). After 1 hour incubation at 37°C, the binding reactions were analyzed by electrophoresis in a 0.8% agarose/Tris-Borate gel without EDTA. The gel was first scanned with a ChemiDoc MP imaging system (BioRad, software Image lab vs 4.1) to detect Alexa Fluor labeled hRAD51 proteins (bottom panels). Next, the gel was stained with ethidium bromide (EtBr) to detect DNA (top panels). Lanes a = DNA alone; lanes b, c, d = unlabeled wild-type hRAD51; lanes f, g, h = hRAD51 K313 C319S labeled with AlexaFluor 555. Lanes e, i = unlabeled wild-type and labeled C319S hRAD51 proteins at 2.5 µM without DNA. NC = Nicked circles; CCC = Covalently closed circles; AF = Alexa Fluor.

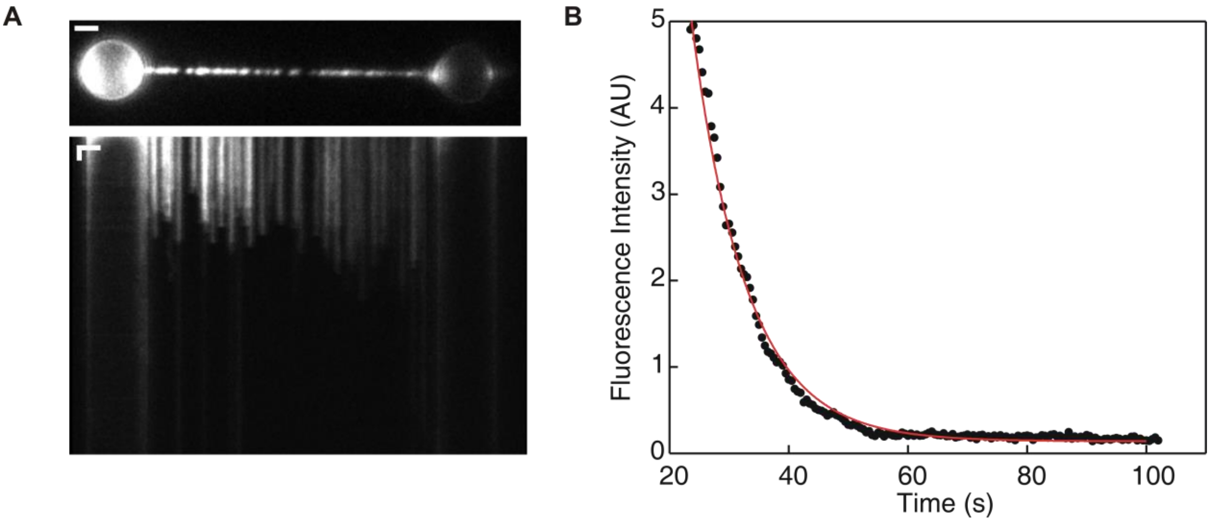

Appendix Figure 2: Photobleaching of hRAD51

A Fluorescence images and kymograph of a hRAD51-ssDNA construct under continuous illumination. Images are a typical example of the results of 24 identical experiments. Scale bars: 2 µm (horizontal) and 5 s (vertical).

B Fluorescence intensity over time of the same hRAD51-ssDNA construct. From exponential fits to such datasets and the datasets taken with images acquired every 30 s, the photobleaching rate can be determined. In this example, the photobleaching rate is $(108 \pm 1) \cdot 10^{-3}\ s^{-1}$. Note that all disassembly experiments are done with a factor of 60 less fluorescence exposure, making the effective photobleaching rate under these imaging conditions $(1.80 \pm 0.02) \cdot 10^{-3}\ s^{-1}$. Images are a typical example of the results of 24 identical experiments.

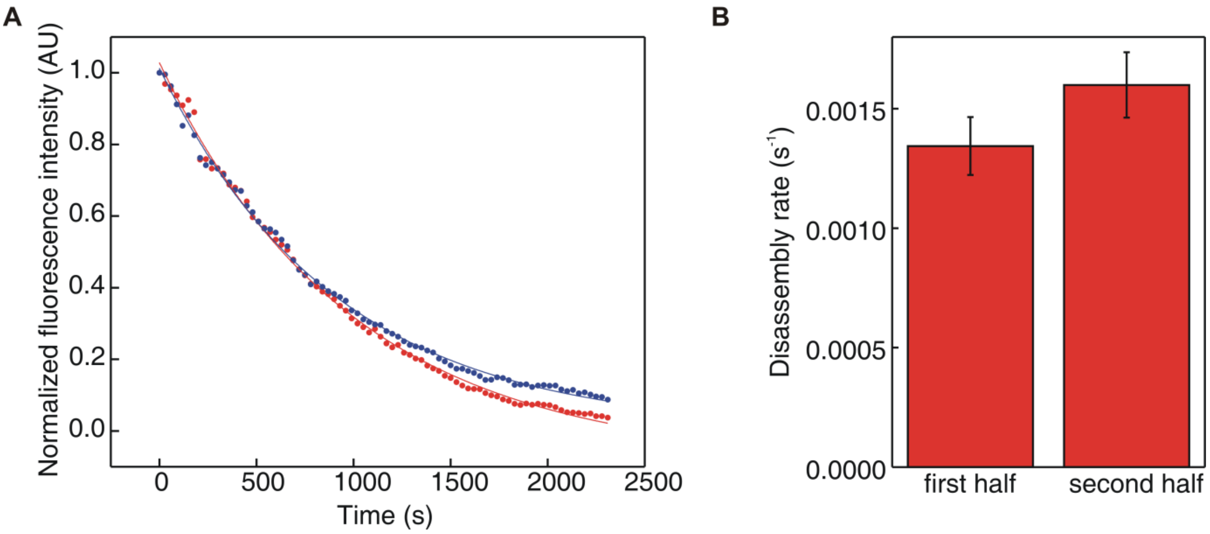

Appendix Figure 3: Disassembly of hRAD51 from ssDNA is sequence-independent

A Normalized integrated fluorescence intensity of two halves of a single hRAD51-ssDNA complex over time and corresponding exponential fits, showing no significant difference between the disassembly rates in both halves. Typical example out of 16 identical experiments.

B For each molecule, the disassembly rate was determined with an exponential fit such as in A. For all molecules, the lowest rate of the two was defined to be the first half, and the highest of the two rates was defined to be the second half. Averaging all first halves and second halves shows no significant difference in average disassembly rate. Since the DNA template used is GC-rich in one half, and AT-rich in the other half, this experiment effectively shows that there is no difference in disassembly from a GC-rich or an AT-rich template. We thus conclude that there is no strong sequence-dependence of hRAD51 disassembly from ssDNA.

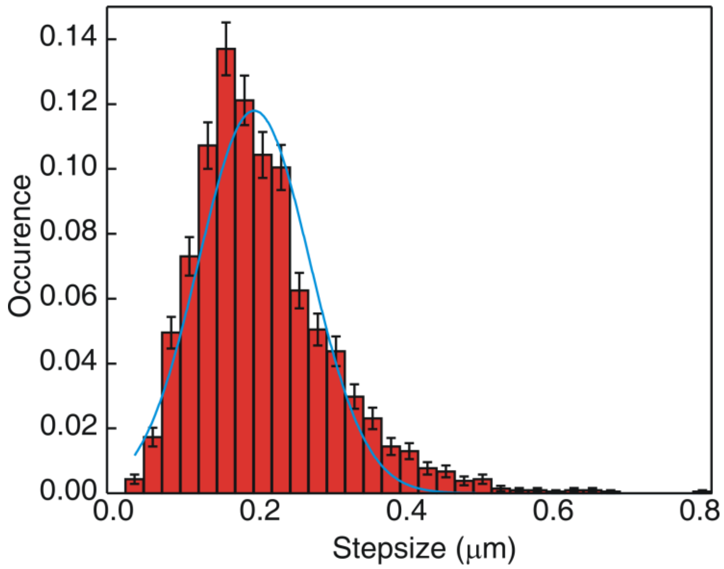

Appendix Figure 4: Distribution of contour length step sizes. Using Kerssemakers[1] step-fitting algorithm, the contour length curves such as shown in Figure 3 can be fit. In total, 2080 steps were detected with an average step size of 200±5 nm.

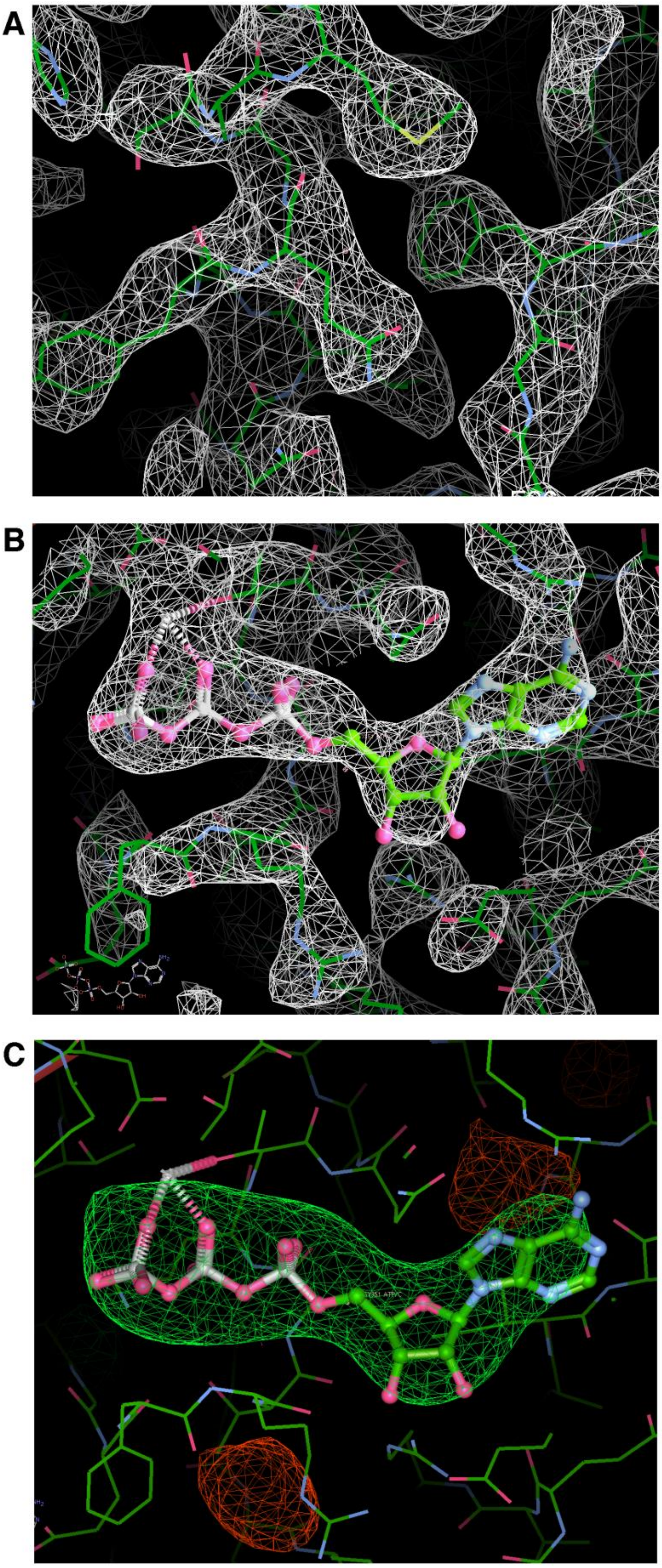
A
B
C

Appendix Figure 5: Details of the electron density map for the crystal structure of the human RAD51-ATP filament. The figure was produced in Coot[2].

A A view of the RAD51 protomer-protomer interface, showing the sharpened 2Fo-Fc electron density map for the crystal structure of the human RAD51-ATP filament, superimposed on the refined crystallographic model. Amino acid F86, which plays a critical role in RAD51 self-association, is labelled. The map is drawn as a white mesh, contoured at 1.3 rmsd; the crystal structure is shown as a stick model, with carbon atoms in green.

B A representative view of the sharpened 2Fo-Fc electron density map for an ATP molecule, bound at the interface between RAD51 protomers B and C in the crystal structure.

C Omit map for the same ATP molecule, calculated in Phenix, using Polder[3]. The density is contoured at 3.0 rsmd and coloured green (positive) or red (negative).

Wavelength (Å)	0.95373
Resolution range (Å)	49.14 - 3.93 (4.07 - 3.93)
Space group	P 1 21 1
Unit cell	117.7Å 128Å 230.1Å 90 90.3 90
Unique reflections	61092 (6038)
Multiplicity	3.8
Completeness (%)	99.83 (99.37)
Mean I/sigma(I)	3.8 (0.5)
Wilson B-factor	128.32
R-merge	0.287 (2.888)
R-meas	0.334 (3.353)
R-pim	0.168 (1.683)
$CC_{1/2}$	0.991(0.263)
Reflections used in refinement	61077 (6038)
Reflections used for R-free	3098 (328)
R-work	0.2684 (0.3601)
R-free	0.3166 (0.3820)
Number of non-hydrogen atoms	32767
macromolecules	32319
ligands	448
Protein residues	4235
RMS(bonds)	0.001
RMS(angles)	0.43
Ramachandran favored (%)	96.24
Ramachandran allowed (%)	3.66
Ramachandran outliers (%)	0.10
Rotamer outliers (%)	0.00
Clashscore	3.39
Average B-factor ($Å^2$)	135.74
macromolecules	135.81
ligands	131.24

Appendix Table 1: Data collection and refinement statistics. Statistics for the highest-resolution shell are shown in parentheses.

Observed disassembly rate	Number of experiments	Experimental conditions	Corresponding figure
$(14\pm2)\cdot10^{-4}\ s^{-1}$	8	20 mM Tris pH 7.5, 100 mM KCl, 1 mM $MgCl_2$, 1 mM ATP, 10 mM DTT / 5 pN	Fig 2
$(15\pm1)\cdot10^{-4}\ s^{-1}$	16	20 mM Tris pH 7.5, 100 mM KCl, 1 mM $MgCl_2$, 1 mM ATP, 10 mM DTT / 20 pN	Fig 2
$(12\pm1)\cdot10^{-4}\ s^{-1}$	4	20 mM Tris pH 7.5, 100 mM KCl, 1 mM $MgCl_2$, 1 mM ATP, 10 mM DTT / 50 pN	Fig 2
$(9\pm2)\cdot10^{-4}\ s^{-1}$	11	20 mM Tris pH 7.5, 100 mM KCl, 1 mM $MgCl_2$, 1 mM ADP, 10 mM DTT	Fig EV3
$(11\pm2)\cdot10^{-4}\ s^{-1}$	16	20 mM Tris pH 7.5, 100 mM KCl, 1 mM $MgCl_2$, 1 mM ATP, 10 mM DTT	Fig EV3
$(4.2\pm0.3)\cdot10^{-4}\ s^{-1}$	2	20 mM Tris pH 7.5, 10 mM $Mg(OAc)_2$, 2 mM $CaCl_2$, 2 mM ADP, 1 mM DTT	Fig 3H
$(8\pm1)\cdot10^{-4}\ s^{-1}$	5	20 mM Tris pH 7.5, 1 mM $MgCl_2$, 1 mM ADP and 10 mM DTT	Fig EV4
$(8\pm1)\cdot10^{-4}\ s^{-1}$	11	20 mM Tris pH 7.5, 100 mM KCl, 1 mM $MgCl_2$, 1 mM ADP, 10 mM DTT	Fig EV4
$(7\pm2)\cdot10^{-4}\ s^{-1}$	3	20 mM Tris pH 7.5, 100 mM KCl, 10 mM $MgCl_2$, 1 mM ADP, 10 mM DTT	Fig EV4
$(3.5\pm0.3)\cdot10^{-4}\ s^{-1}$	6	20 mM Tris pH 7.5, 10 mM $Mg(OAc)_2$, 2 mM $CaCl_2$, 2 mM ATP, 1 mM DTT	Fig EV7B
$(4\pm1)\cdot10^{-4}\ s^{-1}$	6	20 mM Tris pH 7.5, 10 mM $Mg(OAc)_2$, 2 mM $CaCl_2$, 2 mM ATP, 1 mM DTT	Fig EV7C
$(4\pm1)\cdot10^{-4}\ s^{-1}$	1	20 mM Tris pH 7.5, 2 mM $CaCl_2$, 10 mM $Mg(OAc)_2$, 1 mM DTT	Fig EV7E
$(4\pm1)\cdot10^{-4}\ s^{-1}$	1	20 mM Tris pH 7.5, 2 mM $CaCl_2$, 10 mM $Mg(OAc)_2$, 1 mM DTT	Fig EV7F

Appendix Table 2: Overview of RAD51 disassembly rates from ssDNA. Overview of all measured RAD51 disassembly rates under different experimental conditions.
