# Supplementary material for: Two distinct conformational states define the interaction of human RAD51‐ATP with single‐stranded DNA
Source: EMBO J. 2018 Mar 5;37(7):e98162. doi: 10.15252/embj.201798162 (PMC5881629; doi:10.15252/embj.201798162)
Supplement: Supplementary file 2 — Expanded View Figures PDF [file EMBJ-37-e98162-s002.pdf]

Expanded View Figures

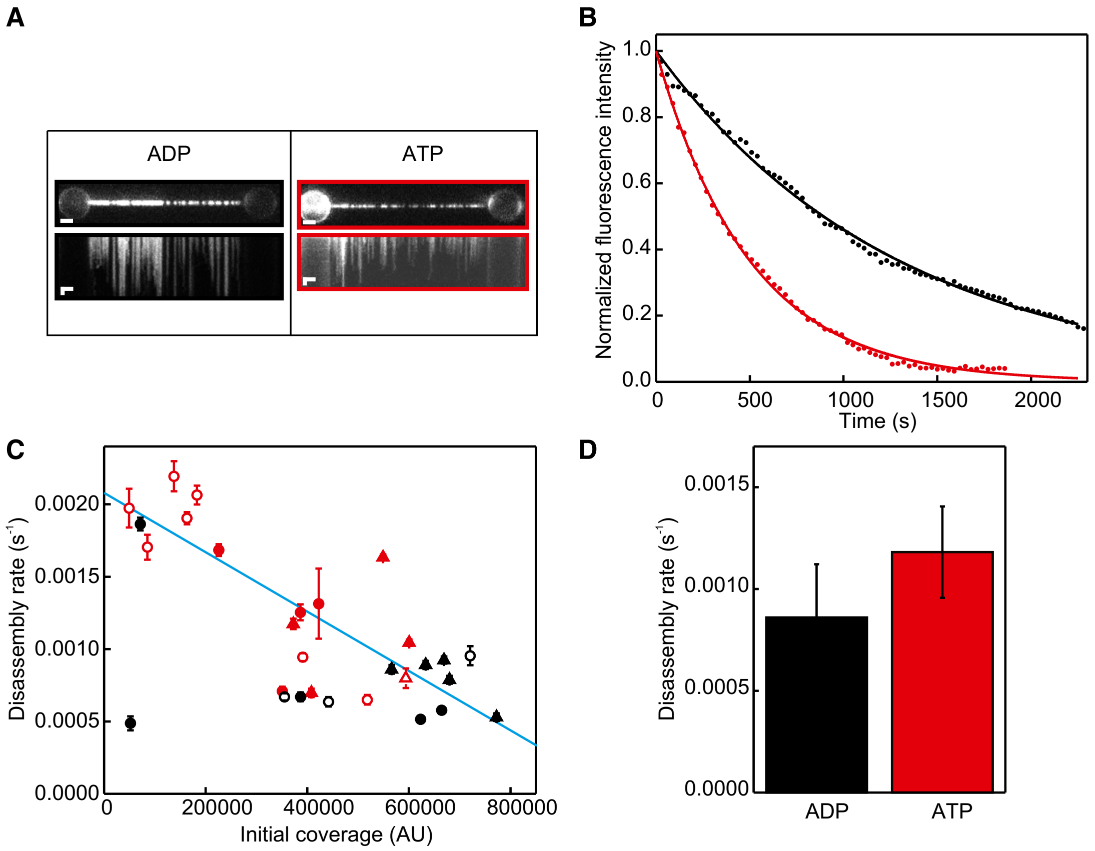

Figure EV1. Disassembly of hRAD51 from ssDNA does not depend on the presence of ATP or ADP in the filament.

A Fluorescence images and kymographs of hRAD51 disassembling from ssDNA for filaments assembled in 20 mM Tris pH 7.5, 40 mM KCl, 10 mM $Mg(OAc)_2$, 2 mM ATP and 10 mM DTT and disassembled in 20 mM Tris pH 7.5, 100 mM KCl, 1 mM $MgCl_2$, 1 mM ATP and 10 mM DTT (right panel) or filaments assembled in 20 mM Tris pH 7.5, 40 mM KCl, 10 mM $Mg(OAc)_2$, 2 mM ADP and 10 mM DTT and disassembled in 20 mM Tris pH 7.5, 100 mM KCl, 1 mM $MgCl_2$, 1 mM ADP and 10 mM DTT (right panel). Images are typical examples of 16 (for the ATP condition) and 11 (for the ADP condition). Scale bars: 2 μm (horizontal) and 5 s (vertical).

B Normalized integrated fluorescence intensity of the images shown in (A) over time. Exponential fits to these traces give disassembly rates of $(6.7 \pm 0.3)\ 10^{-4}\ s^{-1}$ (black dataset; in the presence of ATP) and $(17 \pm 4)\ 10^{-4}\ s^{-1}$ (red dataset; in the presence of ADP). Coloured edges in (A) show colour of corresponding force curve.

C Disassembly rate is correlated with the initial coverage of the DNA molecule. Red closed circles: measured in ATP at 5 pN; red open circles: measured in ATP at 20 pN; red closed triangles: measured in ATP at 50 pN; red open triangles: measured in ATP at 75 pN; black closed circles; measured in ADP in 100 mM KCl and 1 mM $MgCl_2$; black open circles; measured in ADP in 100 mM KCl and 10 mM $MgCl_2$; black triangles: measured in 1 mM $MgCl_2$. Blue line: linear fit with a slope of (-21 ± 3) $10^{-10}\ s^{-1}$/AU (Pearson's correlation coefficient of the fit: −0.84) that was used to correct observed disassembly rates for differences in initial coverage.

D Average disassembly rates in ATP or ADP conditions after correcting for differences in initial coverage. Since the error bars of the black and red datasets overlap, there is no significant difference between the disassembly rate in the presence of ATP or ADP. All coverages were scaled to 400,000 using a linear approximation based on the fit in (C).

Data information: Error bars: SEM, based on 11 molecules (black dataset) and 16 molecules (red dataset).
Source data are available online for this figure.

 © 2018 The Authors

 © 2018 The Authors

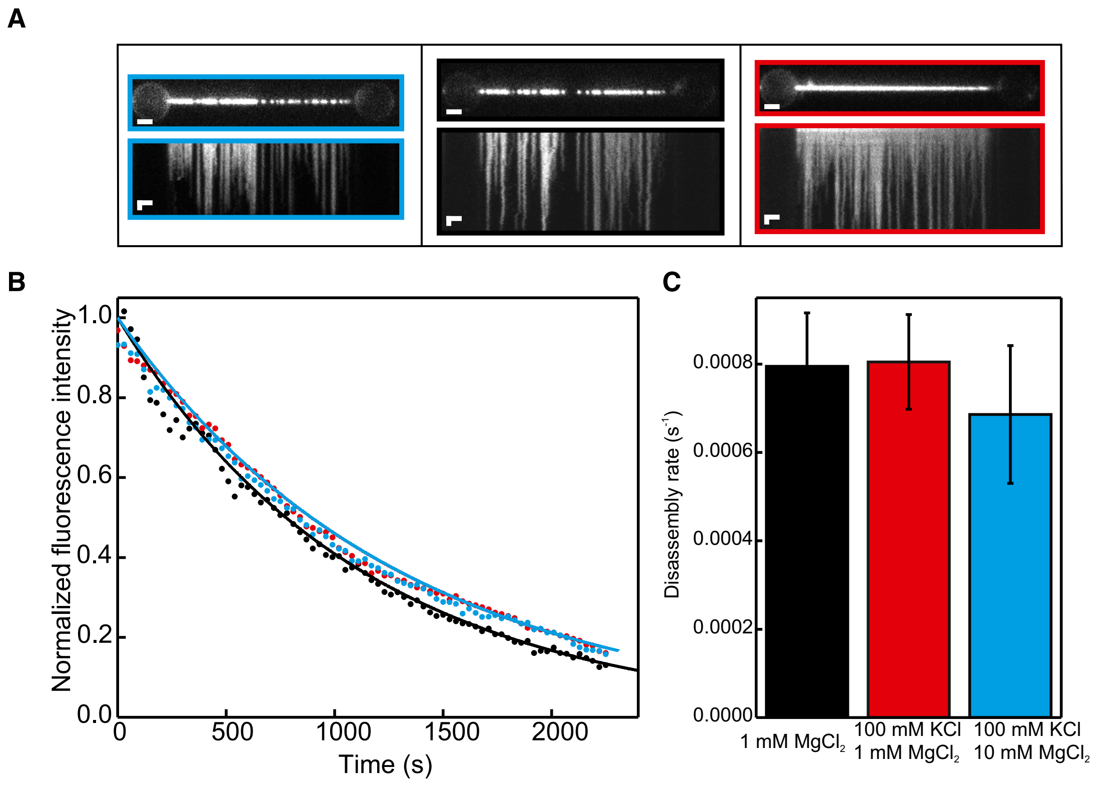

Figure EV2. Disassembly of hRAD51 from ssDNA is independent of ionic strength.

A Fluorescence images and kymographs of hRAD51 disassembling from ssDNA of filaments assembled in 20 mM Tris pH 7.5, 40 mM KCl, 10 mM $Mg(OAc)_2$, 2 mM ADP and 10 mM DTT and disassembled in 20 mM Tris pH 7.5, 1 mM $MgCl_2$, 1 mM ADP and 10 mM DTT (left panel); filaments assembled in 20 mM Tris pH 7.5, 40 mM KCl, 10 mM $Mg(OAc)_2$, 2 mM ADP and 10 mM DTT and disassembled in 20 mM Tris pH 7.5, 100 mM KCl, 1 mM $MgCl_2$, 1 mM ADP and 10 mM DTT (middle panel); and filaments assembled in 20 mM Tris pH 7.5, 40 mM KCl, 10 mM $Mg(OAc)_2$, 2 mM ADP and 10 mM DTT and disassembled in 20 mM Tris pH 7.5, 100 mM KCl, 10 mM $MgCl_2$, 1 mM ADP and 10 mM DTT (right panel). Images are typical examples of 5, 11 and 3 identical experiments, respectively. Scale bars: 2 μm (horizontal) and 5 s (vertical).

B Normalized integrated fluorescence intensity of the images shown in (A) over time. Exponential fits to these traces give disassembly rates of (8 ± 1) 10^{-4} s^{-1} (black dataset), (6.7 ± 0.3) 10^{-4} s^{-1} (red dataset) and (6.7 ± 0.2) 10^{-4} s^{-1} (blue dataset). Coloured edges in (A) show colour of corresponding force curve. Curves are typical examples of 5, 11 and 3 identical experiments, respectively.

C Average disassembly rates at indicated ionic strengths do not vary significantly. Error bars: SEM, based on 5, 11 and 3 identical experiments, respectively.

Source data are available online for this figure.

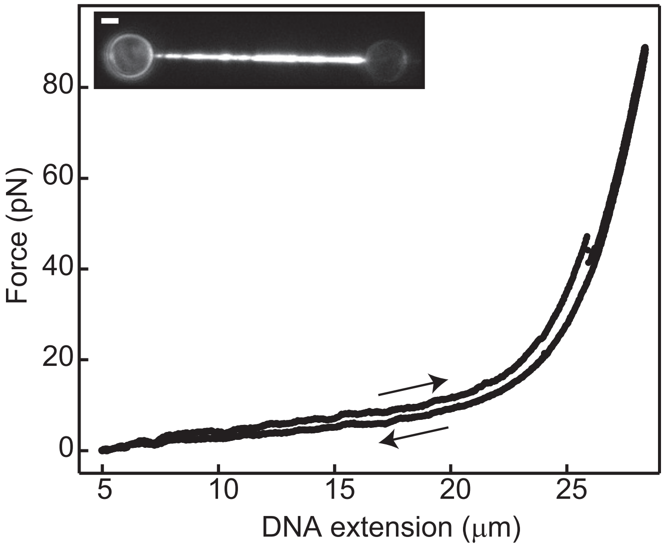

Figure EV3. Structural transitions in ATP and Mg^{2+}.

Force-extension and force-relaxation curve measured in a buffer containing ATP and Mg^{2+}. Under these conditions, ATP hydrolysis and NPF disassembly can occur. We observe a slight hysteresis between extension and relaxation curves. However, the disassembly rate under these conditions is relatively high, such that the assumption that the amount of hRAD51 bound remains constant during one extension–relaxation cycle is no longer valid. Therefore, under these conditions, no quantitative analysis (such as shown for other conditions in Fig 4) of the hysteresis and the structural transitions can be performed. Scale bar: 2 μm.

Source data are available online for this figure.

 © 2018 The Authors

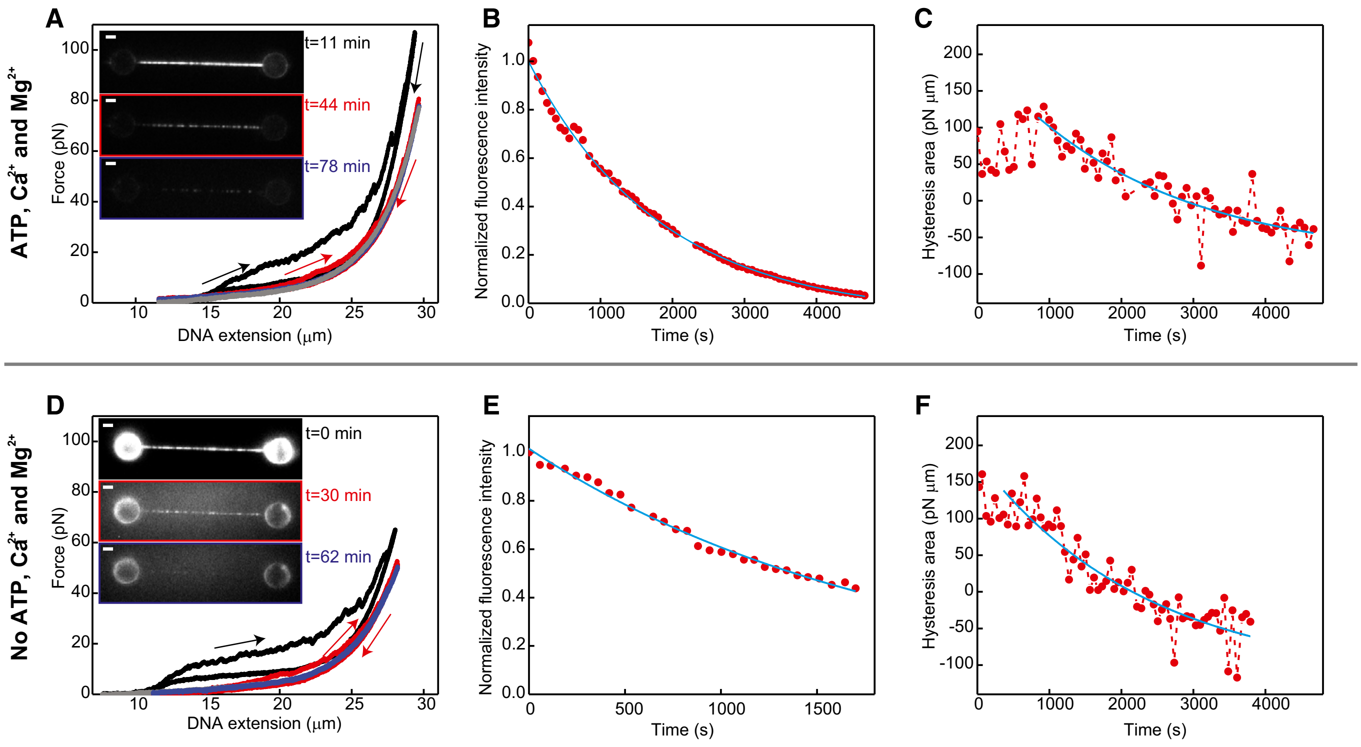

Figure EV4. Structural transitions under different ATP/ADP conditions.

A–C Same as Fig 3A–C but recorded in the presence of ATP, Ca^{2+} and Mg^{2+} (buffer composition: 20 mM Tris pH 7.5, 10 mM $Mg(OAc)_2$, 2 mM $CaCl_2$, 2 mM ATP, 1 mM DTT). Under these conditions, ATP hydrolysis can occur, and thus, hRAD51 can disassemble from the ssDNA. Therefore, the difference between the extension and relaxation curves (A), the total fluorescence intensity (B) and hysteresis area (C) decrease over time. In (A), the blue curve is indistinguishable from the grey curve of bare ssDNA. The disassembly rate can be determined either by an exponential fit to the fluorescence data (B), yielding, after correcting for photobleaching, a rate of $(3.5 \pm 0.3)\ 10^{-4}\ s^{-1}$, or by an exponential fit to the hysteresis data (C), yielding a rate of $(4 \pm 1)\ 10^{-4}\ s^{-1}$. Data shown is a representative example of six identical experiments.

D–F Same experiments as in (A–C) in the presence of Ca^{2+} and Mg^{2+} (20 mM Tris pH 7.5, 2 mM $CaCl_2$, 10 mM $Mg(OAc)_2$, 1 mM DTT). NPFs were formed in the presence of ATP, but there was no ATP or ADP in the observation channel. Under these conditions, ATP hydrolysis can occur, and thus, hRAD51 can disassemble from the ssDNA, but reloading of ATP to the NPF after ATP hydrolysis and ADP release is impossible. The differences between the extension and relaxation curves (D), the total fluorescence intensity (E) and hysteresis area (F) decrease over time. In (D), blue curve is indistinguishable from grey curve of bare ssDNA. The disassembly rate can be determined by an exponential fit to the data in (E), giving a rate of $(4 \pm 1)\ 10^{-4}\ s^{-1}$, or by an exponential fit to the data in (F), giving a rate of $(4 \pm 1)\ 10^{-4}\ s^{-1}$.

Source data are available online for this figure.

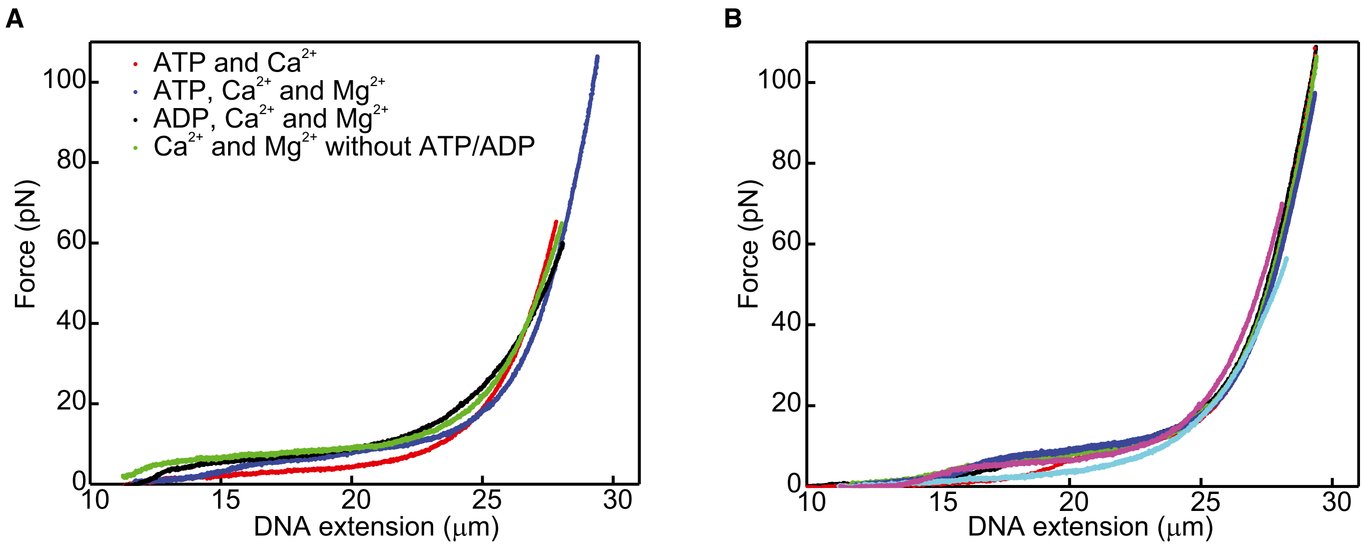

Figure EV5. Lengths of the different hRAD51-ssDNA NPF states.

A Force-relaxation curves under different experimental conditions (see text and Fig 3 for details on conditions). Since the mechanical properties of the ADP-bound filaments (black curve) do not deviate significantly from the other conditions (red, blue and green curves), we assume that the length of the ADP-bound filament is comparable to that of the ATP-bound conformations.

B For comparison, six individual force-relaxation curves measured under the same conditions (ATP, Ca^{2+} and Mg^{2+}) are shown. Variation between curves in (A) is in the same order as the variation between the curves in (B), showing that the variation in (A) is caused by molecule-to-molecule variation and not by the different experimental conditions.

Source data are available online for this figure.

 © 2018 The Authors
