# Supplementary material for: Two distinct conformational states define the interaction of human RAD51‐ATP with single‐stranded DNA
Source: EMBO J. 2018 Mar 5;37(7):e98162. doi: 10.15252/embj.201798162 (PMC5881629; doi:10.15252/embj.201798162)
Supplement: Supplementary file 3 — Movie EV1 [file EMBJ-37-e98162-s003.zip › Movie_EV1.docx]

**Movie EV1:** The movie illustrates a side view of the conformational change that might take place in the filament when RAD51 protomers undergo a concerted transition between the two inter-subunit arrangements observed in the crystal structure of the human RAD51-ATP filament structure. The movie was produced in Chimera, morphing between RAD51 filaments that contain exclusively one or the other inter-protomer arrangements. The ATP ligands are not shown.
