# Supplementary material for: Two distinct conformational states define the interaction of human RAD51‐ATP with single‐stranded DNA
Source: EMBO J. 2018 Mar 5;37(7):e98162. doi: 10.15252/embj.201798162 (PMC5881629; doi:10.15252/embj.201798162)
Supplement: Supplementary file 4 — Movie EV2 [file EMBJ-37-e98162-s004.zip › Movie_EV2.docx]

**Movie EV2:** Top view of the RAD51-ATP filament transition shown in Appendix movie 1.
